# Zymosan attenuates melanoma growth progression, increases splenocyte proliferation and induces TLR-2/4 and TNF-α expression in mice

**DOI:** 10.1186/s12950-018-0182-y

**Published:** 2018-03-22

**Authors:** Mehdi Taghavi, Esmaeil Mortaz, Alireza Khosravi, Ghasem Vahedi, Gert Folkerts, Mohammad Varahram, Mehdi Kazempour-Dizaji, Johan Garssen, Ian M. Adcock

**Affiliations:** 1 Mycology Research Center, Faculty of Veterinary Medicine, University of Tehran, Tehran, Iran; 2 Department of Immunology, Faculty of Medicine, ShahidBeheshti University of Medical Sciences, Tehran, Iran; 3 Division of Pharmacology, Utrecht Institute for Pharmaceutical Sciences, Faculty of Sciences, Utrecht University, Utrecht, the Netherlands; 4 Clinical Tuberculosis and Epidemiology Research Center, National Research Institute of Tuberculosis and Lung Diseases (NRITLD), Shahid Beheshti University of Medical Sciences, Tehran, Iran; 5 Mycobacteriology Research Center (MRC), National Research Institute of Tuberculosis and Lung Diseases (NRITLD), Shahid Beheshti University of Medical Sciences, Tehran, Iran; 6 Nutricia Research Centre for Specialized Nutrition, Utrecht, Netherlands; 7 Airways Disease Section, National Heart and Lung Institute, Faculty of Medicine, Imperial College London, London, UK

**Keywords:** TLR-2, TLR-4, TNF-α, Zymosan

## Abstract

**Background:**

Melanoma is one of the most common types of skin malignancies. Since current therapies are suboptimal, considerable interest has focused on novel natural-based treatments. Toll-like receptors (TLRs) play an important role in evoking innate immunity against cancer cells. Zymosan, a known TLR-2 agonist, is a glucan derived from yeast cell walls with promising immunomodulatory effects. The aim of this study was to evaluate whether *Saccharomyces cerevisiae*-derived zymosan-modulated skin melanoma progression by regulation of TLR-2 and TLR-4 expression in peritoneal macrophages and serum TNF-α level.

**Methods:**

Male C57BL/6 mice were divided into four groups: i) zymosan-treated (Z), ii) Melanoma-bearing mice (M), iii) Melanoma-bearing mice treated with zymosan (ZM) and iv) a healthy control group (negative control). 15 days after melanoma induction, mice were injected i.p. with zymosan (10 μg) daily for 4 consecutive days. Mice were CO_2_-euthanized and serum TNF-α level, TLR-2 and TLR-4 expression in peritoneal macrophages and tumor growth measured. Splenocytes were treated ex-vivo with zymosan to determine viability and proliferation.

**Results:**

Tumor weight significantly decreased following therapeutic dosing with zymosan (*P* < 0.05). This was associated with zymosan-induced upregulation of TLR-2, TLR-4 and TNF-α mRNA in peritoneal macrophages and enhanced serum TNF-α levels (*P* < 0.05). Splenocyte number and viability were increased in a concentration-dependent manner by zymosan.

**Conclusions:**

Our study suggests that zymosan-induced upregulation of TLR-2, TLR-4 and TNF-α gene expression and of TNF-α release; together with increased level of lymphocyte proliferation may play a role in the inhibition of melanoma progression.

## Background

Melanoma is a skin malignancy which develops from abnormal melanocytes and usually has a low response to conventional therapies [1]. It is a major cause of death in patients with skin cancer and approximately 55,000 deaths from melanoma were reported in 2012 [2]. The prevalence of the disease has increased alarmingly in recent years although the reasons for this are not well understood [3]. Metastatic melanoma has a median survival time of less than 1 year and there is no effective FDA-approved therapy [1]. Melanoma cells usually express self-antigens [4] which are not recognized by the host immune cells. In recent years, our knowledge and understanding of the melanoma has been advanced by the discovery of some of its specific antigens [5].

Multiple pathways are involved in both the priming and effector phases of melanoma rejection. Studies in animal models of melanoma showed a requirement for CD4+ T cells in the priming phase of tumor immunotherapy [6]. Recent attention has focused on drugs that regulate the immune response to cancer cells. For example, cytokines such as IL-2 and IFN-α are used to reduce tumor invasion in the skin [7]. Toll-like receptors (TLRs) recognize pathogen associated molecular patterns (PAMPs) during infection and play a significant role in innate and adoptive immunity of diseases. TLRs are expressed on human cancer cells and TLR-based therapies have been evaluated as anti-cancer immunotherapies [8–10].

For many years, herbal and natural agents have been used for the prevention and treatment of cancers. The interest in natural remedies as anticancer agents has arisen due to the increased number of cancer patients and the significant side effects of synthetic chemotherapy agents [11]. Zymosan is a structural component of yeast cell wall which is rich in β-glucan and mannan and can bind to TLR2 on inflammatory cells. Consequently, it has been used to study the immune responses to TLR2 activation [12, 13]. Moreover, zymosan interacts with dectin-1 receptors on macrophages to stimulate the release of pro-inflammatory mediators and to modulate TLR expression and function [12, 14, 15]. We hypothesized that zymosan would attenuate melanoma growth and so we assessed the therapeutic effect of zymosan in an in vivo mouse model of melanoma. In the current study the effect of Zymosan A derived from *Saccharomyces cerevisiae* on serum TNF-α expression and on TLR2 and 4 genes expression in peritoneal macrophages and on melanoma growth progression was investigated.

## Methods

### Animals and reagents

6–8 week old female C57BL/6 mice (Razi Vaccine and Serum Research Institute, Karaj, Iran, average weight = 18 ± 1.1 g, healthy, drug-naive) were kept at 20 °C with a relative humidity of 55 ± 10% and 12 h light/dark cycles. Mice were randomly divided into 4 groups of 5 animals each:

i) Group Z: healthy mice receiving 10 μg zymosan intraperitoneally (i.p.) (*n* = 5),
ii) Group M: tumor-induced mice receiving normal saline (i.p.) (*n* = 5),
iii) Group ZM: tumor-induced mice receiving 10 μg zymosan i.p. (n = 5),
iv) Group C: healthy mice receiving normal saline (i.p.) as controls (n = 5).

Animals were acclimatized to the environment for 1 week prior to the start of the study. Standard pellet food and tap water were provided ad libitum. For preparation of tumor tissues we had to CO2-euthanized all mice in the current study.

### Tumor induction

The B16F10 melanoma cell line (Pasteur institute, Tehran, Iran) were incubated in RPMI medium (Gibco, Grand Island, NY, USA) containing 10% FBS at 37 °C and 5% CO_2_. Melanoma was induced by subcutaneous injection of 1 × 10^6^ B16F10 cells into the shaved lateral left flank of each mouse. Tumors were successfully induced in all injected mice. All observations were done performed every other day by one observer until establishment of a palpable tumor in each mouse. After successful tumor induction, the measurements were taken every day using the caliper. Tumor sizes never have been exceeded 1.5 cm in diameter according to the IACUC recommendations. In addition, the behavioral and clinical changes in mice such as notable reduction in food uptake, reduced mobility, lethargy and wound or necrosis problems of mice were monitored to exclude any mice exhibiting these characteristics. Regular monitoring of the mice did not reveal any unexpected deaths or severe clinical signs.. In addition to physical examinations we also performed histopathologic evaluations to confirm the establishment of subcutaneous melanoma. Histopathological sections prepared from tumor were investigated by two dermatopathologists. Mitotic rate/index, vascular invasion, lymphocyte infiltration, necrosis and hemorrhage were determined in a blinded manner.

### Zymosan preparation

Zymosan A from *Saccharomyces cerevisiae* (Biosynth International Inc., Itasca, IL, USA) was stored at − 20 °C prior to use. Mice were injected with 10 μg zymosan (groups Z and ZM) daily i.p. for four consecutive days commencing 15 days after tumor-cells were injected.

### Tumor growth

Tumor size was measured by carefully dissecting out and weighting the tumor tissue after CO_2_-induced euthanization of the mice.

### Cell viability and proliferation assay

MTT was used to investigate lymphocyte viability. In brief, splenocytes were isolated from healthy mice. RMPI 1640 medium supplemented with 10% FBS and antibiotics used as culture medium. 1 × 10^5^ cells were seeded into the wells of two 96-well plates containing various concentrations of zymosan (0–100 μg/ml) and were incubated at 37 °C and 5% CO_2_ for 72 h. Cells from one plate were counted using trypan blue. Then, MTT was added into each well of the other plate and incubated for a further 4 h. 150 μl DMSO was added to stop the reaction and the optical density was read at 570 nm using an ELISA plate reader.

### Cytokine measurement

Blood was collected from each mouse by heart puncture. Sera was isolated by centrifugation and the levels of TNF-α measured using a Bio-PlexPro™ Mouse Cytokine, Chemokine and Growth Factor Assay (Bio-Rad, Richmond, CA, USA) according to the manufacturer’s instructions.

### Peritoneal macrophage isolation

At the end of the 4-days treatment with zymosan or saline, mice were euthanized and peritoneal cells harvested by washing the peritoneal cavity with 10 ml of ice-cold toxin-free PBS containing 3% fetal calf serum. Cells were centrifuged at 300×g, 4 °C for 10 min and suspended in 1 ml complete DMEM medium supplemented with 10% FBS. Cells were counted using a haemocytometer and 3 × 10^6^ cells were cultured on 6 well plates at 37 °C, 5% CO_2_ for 2 h to allow macrophages to adhere to the plates. Non-adherent cells were removed by washing the wells with cold sterile PBS. Cell viability was assessed by trypan blue staining. Macrophages were subsequently suspended in DMEM containing 10% FBS.

### RNA isolation and cDNA synthesis

Total RNA was isolated using TRIzol according to the manufacturer’s instructions (Invitrogen, Carlsbad, CA, USA). The quantity and purity of RNA was measured by nanodrop (NanoDrop® ND-1000; NanoDrop Technologies, Wilmington, DE, USA). RNA samples were transcribed to cDNA using an Intron MaximeRT premix kit (Intronbio, South Korea) according to the manufacturer’s instructions. The synthetized cDNA was kept at − 20 °C prior to use.

### Real-time quantitative PCR (RT-qPCR)

Quantitative evaluation of target genes expression was performed using a SYBR Green I based kit (SYBR® Premix Ex Taq™ II TliRNaseH Plus, TaKaRa Bio, Shiga, Japan) in a Rotor-Gene Q real time PCR machine (Qiagen, Hilden, Germany). Previously published primer pairs for target genes and the reference gene are shown in Table 1 [16–19]. 20 μl reaction mixtures were prepared in thin-walled PCR tubes as follows for each gene in triplicate: 10 μl TaKaRa qPCR master mix, 2 μl of diluted cDNA template (dilution ratio was 1:10), 0.5 μl of 10 mM forward and reverse primers and 7 μl dH_2_O (sterile distilled water). After initial denaturation at 95 °C for 30 s, 40 cycles [denaturation: 95 °C for 5 s and annealing and extension (60 °C for 30 s)] of PCR was performed. A melting curve analysis was performed using a temperature range between 60 and 92 °C.

**Table 1:** Primer sequences were used at present study

Gene Symbol		Sequence (5′-3′)	Amplicon Size (bp)	Reference
GAPDH	Sense	TGTTCCTACCCCCAATGTGT	138	Lukacs et al. [16]
	Antisense	GGTCCTCAGTGTAGCCCAAG		
TLR2	Sense	CCAAGAGGAAGCCCAAGAAAG	51	Matsushima et al. [17]
	Antisense	AGGCATCATAGCAAACGTCCC		
TLR4	Sense	GGACTCTGATCATGGCACTG	101	Ellett et al. [18]
	Antisense	CTGATCCATGCATTGGTAGGT		
TNF-α	Sense	GCACCACCATCAAGGACTCAA	51	Auerbuch et al. [19]
	Antisense	TCGAGGCTCCAGTGAATTCG		

### Statistical analysis and comparative Ct method

The effect size was predicted to be at least 50% which together with power and significance levels of 95% and 0.05, respectively gave a total sample size of 20 mice (*n* = 5 for each group). Mann–Whitney U test and linear regression analysis were performed using MedCalc version 12 (MedCalc Software, Ostend, Belgium) on the raw data and data presented as means ± S.D. *Alpha* values were set at 0.05 and *P*-values < 0.05 were considered as significant. Bonferroni’s correction was used where multiple comparisons were made. We employed the delta delta Ct (∆∆Ct) method to calculate the relative expression of target genes [20]. The fold change of relative target gene expression was calculated by first normalization of the Ct (threshold cycle) value of the gene of interest relative to reference gene followed by the subtraction of the delta Ct value of treated samples from the delta Ct value of un-treated samples (control group). The data was presented using a log_2_ scale to ensure that similar up or down fold-changes gave a similar change above and below the X axis.

## Results

### Zymosan inhibited melanoma progression

**Fig. 1:**
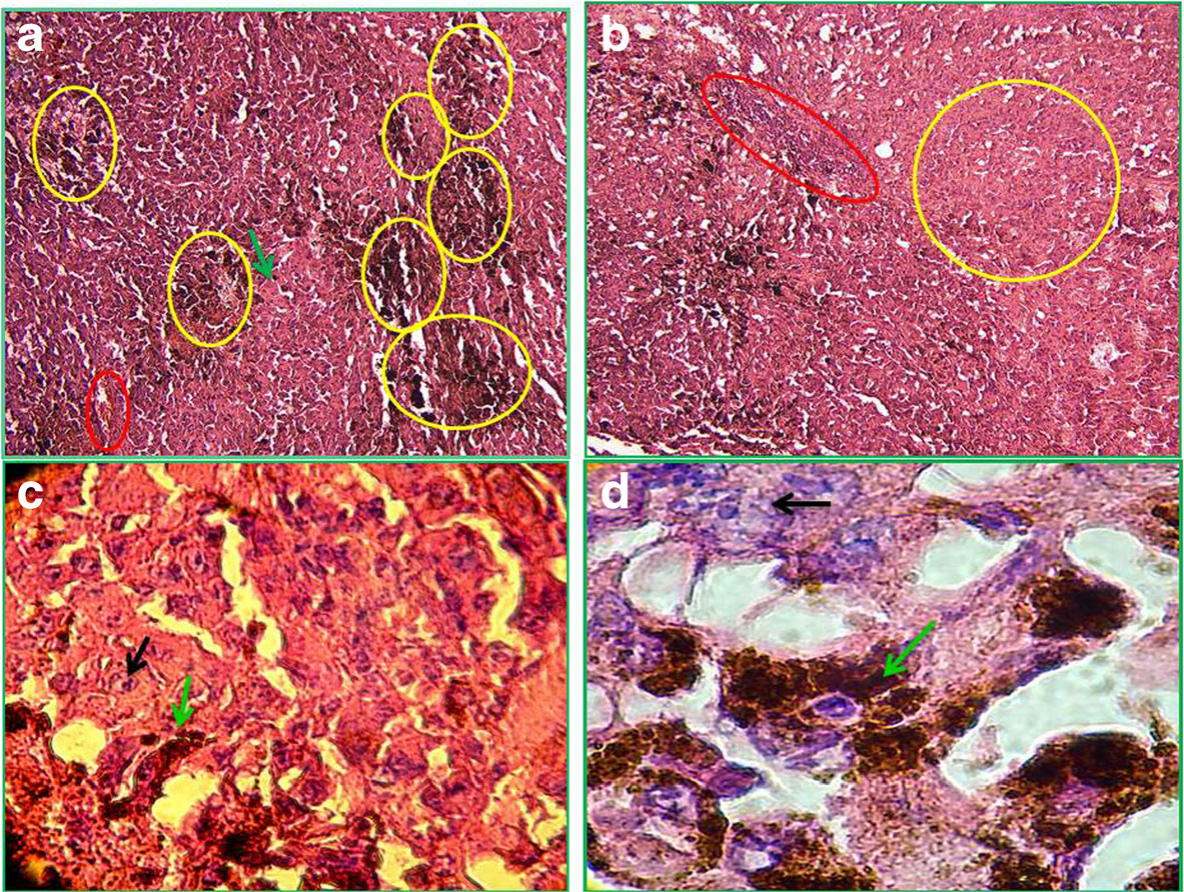
Histopathologic sections obtained from melanoma tumor autopsy in mouse stained with H&E (10–100 x magnifications). Representative histological analysis of tumor tissue from melanoma-bearing mice (*n* = 5). In panel **a** (10× magnification) the yellow circles indicate areas of intensive melanin pigmentation, a red circle indicates an area of hemorrhage and a green arrow highlights an invasive mitotic melanocyte. Its condensed genetic material (chromatin) indicates this as being mitotic. In panel **b** (10× magnification) the yellow circle shows an area containing many mitotic melanocytes and the neighboring red box area shows vascular invasion by some of the invasive melanocytes. In panel **c** (40× magnification) the upper black arrow shows a large mitotic melanocyte (with segmented chromatin and genetic material) and the lower green arrow shows melanin pigmentation (dark brown areas). In panel **d** (100× magnification) the upper black arrow shows a large mitotic melanocyte (with purple-stained genetic material) and the lower green arrow shows melanin pigmentation (dark brown area). All mice showed similar melanoma features

**Fig. 2:**
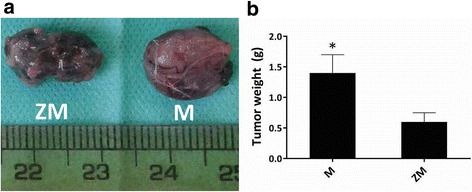
Effect of therapeutic zymosan treatment on melanoma weight and size in melanoma-bearing mice. Representative pictures showing tumor size in melanoma-bearing (M) mice and the effect of therapeutic treatment with zymosan (10 μg, i.p.) in melanoma-bearing (ZM) mice (**a**). The results are presented graphically as means ± SD for all 5 animals in each group (**b**). *P < 0.05 compared with tumor size in M mice. ZM indicates melanoma-bearing zymosan-treated mice

Melanoma growth was confirmed by histopathologic evaluation of the skin tumors of mice (Fig. 1). Moderate pigmentation (yellowish-brown pigments) indicating the presence of melanin was observed in the hematoxylin and eosin (H&E)–stained sections and intense pigmentation areas were observed in the nests of tumor melanocytes (Fig. 1a). Vascular invasion and slight hemorrhage were also observed but no lymphocyte infiltration was found (Fig. 1b). The mean mitotic rate was 11 mitoses/mm^2^ (Fig. 1b). Mitotic morphology of nuclear chromatin and abnormal spindle-shaped invasive melanocytes were also observed in tumor tissue (Fig. 1b and d). The presence of large mitotic melanocytes and of melanin pigmentation is shown at increasingly levels of magnification (Fig. 1c and d) in response to tumor induction. The mean weight of tumor in the zymosan-treated group (group ZM) was significantly lower than in the untreated group (M) (*P* < 0.05) (Fig. 2).

### Zymosan induced mRNA expression and serum levels of TNF-α

**Fig. 3:**
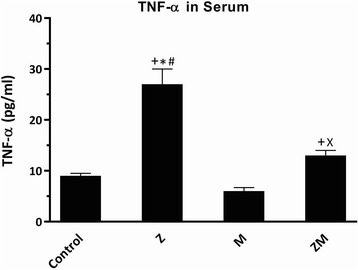
Serum TNF-α level in zymosan-treated (Z) and melanoma-bearing (M) mice. Serum TNF-α measured by ELISA was determined in control untreated mice, M, Z and zymosan-treated (10 μg, i.p.) melanoma-bearing (ZM) mice. The results are presented as means ± SD of 5 mice in each group. ^+^*P* < 0.05 compared with control group, *P < 0.05 compared with M mice, ^#^*P* < 0.05 compared with ZM, ^X^*P* < 0.05 compared with Z mice

Mice in the zymosan group (Z) had significantly greater serum TNF-α than any other group (*P* < 0.05)(Fig. 3). In contrast, serum TNF-α levels in the melanoma-bearing mice (M) were not significantly different from levels seen in control animals (C) (Fig. 3). Serum TNF-α levels in the zymosan-treated melanoma (ZM) group were elevated compared to those observed in the C and M groups (Fig. 3).

**Fig. 4:**
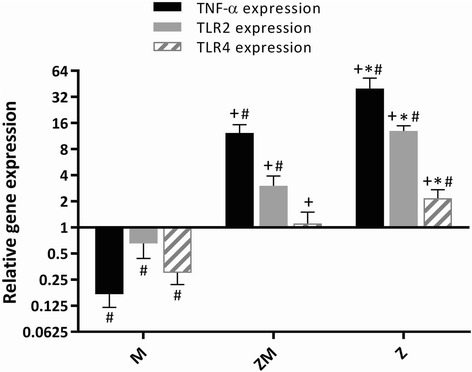
Effect of zymosan treatment (Z) on TNF-α, TLR-2 and TLR-4 genes expression in mouse peritoneal macrophages in melanoma-bearing (M) mice. The expression of TNF-α (black bars), TLR-2 (grey bars) and TLR-4 (lined bars) mRNA in M, Z and zymosan-treated (10 μg, i.p.) melanoma-bearing (ZM) mice was determined relative to that in control mice. Results are presented as means ± SD of n = 5 animals per group. ^+^*P* < 0.05 compared with M mice, *P < 0.05 compared with ZM mice, ^#^P < 0.05 compared with control mice

TNF-α mRNA levels in peritoneal macrophages from melanoma-bearing mice (M) were decreased compared to control animals but were significantly elevated in zymosan-treated control animals (Z) and in zymosan-treated melanoma-bearing animals (ZM) (all *P* < 0.05)(Fig. 4, Tables 2 and 3). The expression of TNF-α mRNA in peritoneal macrophages from ZM animals was intermediate between that in Z and M animals with a significant difference in expression between values from the M and Z alone groups.

**Table 2:** Significance values of TNF-α, TLR-2 and TLR-4 genes expression between groups

Comparator groups	Target genes		
	TNF-α	TLR-2	TLR-4
M/C	0.002	0.0055	0.002
ZM/C	0.0018	0.0019	0.137
Z/C	0.0017	0.0019	0.002
M/ZM	0.0017	0.00197	0.00196
M/Z	0.00167	0.0018	0.00194
ZM/Z	0.00196	0.00197	0.0025

*C* control untreated mice, *M* melanoma-bearing mice, *Z* zymosan-treated mice, *ZM* zymosan-treated melanoma-bearing mice. *P* values are reported as Mann-Whiney U tests

**Table 3:** Fold change values of TNF-α, TLR-2 and TLR-4 genes expression relative to control group

Groups	Target genes		
	TNF-α	TLR-2	TLR-4
M	0.17 ± 0.05	0.66 ± 0.22	0.3 ± 0.08
ZM	12.27 ± 3.07	3.02 ± 0.9	1.11 ± 0.4
Z	39.85 ± 12.75	12.95 ± 1.94	2.18 ± 0.54

*M* melanoma-bearing mice, *Z* zymosan-treated mice, *ZM* zymosan-treated melanoma-bearing mice. Data were presented as means ± standard deviation

### Zymosan upregulated TLR-2 and TLR-4 gene expression

Melanoma growth was associated with a significant reduction in TNF-α, TLR-2 and TLR-4 mRNA expression in peritoneal macrophages compared to that in control mice (*P* < 0.05). TNF-α mRNA expression in melanoma-bearing mice (M) was reduced by 83% compared to control mice while TLR-4 and TLR-2 mRNA expression was suppressed by 70% and 35%, respectively compared to control mice (Fig. 4, Tables 2 and 3). In contrast, zymosan significantly induced TNF-α and TLR-2 mRNA expression in both healthy (Z) animals and in melanoma-bearing (ZM) animals. The expression of TLR-2 mRNA in the ZM mice (Fig. 4, Tables 2 and 3) was intermediate between that in the Z and M animals. The mRNA level of TLR-4 in Z and M mice followed the same pattern seen for TNF-α and TLR2 (Fig. 4, Tables 2 and 3). TLR4 mRNA expression in the ZM mice was no different from that seen in control mice being intermediate between that seen in M and Z mice (Fig. 4, Tables 2 and 3).

### Zymosan stimulates splenocyte proliferation

**Fig. 5:**
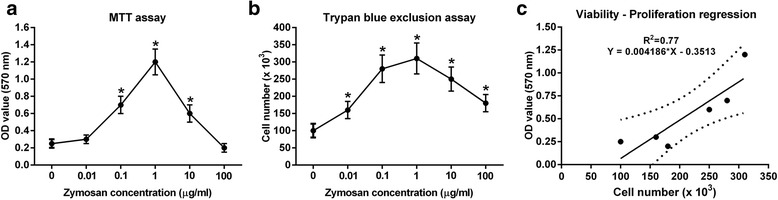
The viability and proliferation of murine splenocytes ex-vivo is increased concentration-dependently by zymosan treatment. Panel **a** represents the viability of splenocytes after 72 h treatment with different concentrations of zymosan as determined by MTT assay. Panel **b** represents number of splenocytes as measured by trypan blue exclusion assay when treated for 72 h with increasing concentrations of zymosan. Panel **c** represents the correlation between splenocyte viability and proliferation. The data are reported as the means ± SD of at least 3 independent experiments. *P < 0.05 compared with control splenocytes not treated with zymosan

Zymosan induced a concentration-dependent increase in splenocyte viability (Fig. 5a) and proliferation (Fig. 5b). Splenocyte viability in the 0.1, 1 and 10 μg/ml zymosan-treated cells was significantly higher than in the control media-treated cells (*P* < 0.05). The viability reached a maximum at 1 μg/ml zymosan treatment before being reduced at the highest concentrations tested. The viability at a high concentration of zymosan (100 μg/ml) tested was similar to that observed in the control (untreated) cells (Fig. 5a). Similar results were observed with the splenocyte proliferation assays. Zymosan, at all concentrations, induced splenocyte proliferation compared to negative control cells (*P* < 0.05). However, at the higher zymosan concentrations of 10 and 100 μg/ml splenocyte proliferation was decreased compared to the maximal effect seen at 1 μg/ml (Fig. 5b). There was a strong positive correlation between viability and proliferation rates of splenocytes (linear regression, R^2^ = 0.77) (Fig. 5c).

## Discussion

This study demonstrated that therapeutic treatment with zymosan attenuated melanoma growth in vivo. This therapeutic response was associated with an up-regulation of TNF-α and TLR-2/4 gene expression along with splenocyte viability and proliferation. We hypothesize that these immunomodulatory events are associated with zymosan-induced tumor inhibition.

The major constituent of zymosan is β-glucan which is responsible for anti-tumor activity of zymosan [21]. Tumor-inhibition by oxidized-zymosan (OX-ZYM), which is mainly composed of 1,3-β- and 1,6-β-glucan was significantly greater than that achieved with reduced and hydrolyzed OX-ZYM in which its 1,6-β-glucan moiety was removed. This indicates the central role of (1,6)-branched (1,3)-β-glucans in inhibiting tumor growth [21].

The antitumor effect of zymosan was first studied in sarcoma-bearing mice in 1957 by Bradner et al., who reported that zymosan promoted tumor depletion by utilizing a host defense response [22]. Subsequent studies have confirmed that the major anti-tumor activities of glucans are due to their effects on the immune system rather than a direct effect on tumor cells. Our results also showed a considerable anti-tumor effect when administering zymosan therapeutically to melanoma-bearing mice. The mean tumor weight being significantly reduced by ~ 50% in zymosan-treated melanoma-bearing mice compared to non-treated control mice. Future studies will examine the potential of zymosan co-treatment with other anti-cancer agents to completely suppress melanoma survival.

A previous study reported that zymosan (100 μg/mL) could induce a 300% greater production of TNF-α from murine macrophages compared to that elicited by LPS (100 ng/mL) [14]. Furthermore, a synthetic β-(1 → 3) gluco-hexose drives macrophages towards an M1-like phenotype and enhances the production of numerous inflammatory cytokines such as IL-1α, IL-1β, IL-12, IL-16, IL-17, IL-23, TNF-α and IFN-γ [23]. β-(1,3)-(1,6)-glucan derived from baker’s yeast also could induce similar levels of TNF-α, IL-8 and IL-6 production from whole blood cells as seen with LPS [24]. These observations are in agreement with our previous study, where zymosan promoted the expression of IL-6 and IL-1β in melanoma-bearing mice and enhanced the phagocytic ability of macrophages [13].

Moreover, in the present study, TNF-α was strongly up-regulated in zymosan-administered mice in comparison to other experimental groups at both the protein and mRNA level. TNF-α protein production and release can be due to strong induction of TLR-2 by zymosan stimulation. This was associated with a significant reduction in tumor weight indicating an impact of zymosan on the immune response against tumor cells. The precise mechanism for this effect is unclear although in a previous study it was reported that oral zymosan treatment of subcutaneous S180 tumor-bearing mice enhanced the immune response via having a significant antioxidant effect [25]. Further studies utilizing TNF-α cell-specific conditional knockout mice or anti-TNF mAbs will address the exact role of TNF-α in immune cell activation and tumor suppression in this model.

TLR2 and dectin-1, the major zymosan receptors [14], act co-operatively to detect microbial PAMPs [26, 27, 28]. Macrophages recognize β-glucan using TL2/4 and dectin-1 [23] and zymosan, acting through TLR2/4, can induce a similar level of NF-κB/AP-1 activity in HEK cells as seen with specific TLR-2 (Pam3CSK4) and TLR4 (LPS) ligands [14]. Zymosan (≤100 μg/mL) induces NF-κB/AP-1-related TLR-2 and TLR4 activity [14]. In our study we detected a significant increase in TLR4 mRNA in macrophages from zymosan-treated mice. In contrast, there is also a significant reduction in TLR-4 expression in melanoma-bearing mice, indicating a strong suppressor effect of melanoma on its level. The positive effect of zymosan on TLR-4 regulation could be due to two reasons: the feedforward effect of cytokines on TLR-4 (e.g. positive effect of IFN-γ on TLR-4) and activation of macrophages. It was known that some cytokines promote TLR expression. IFN-γ shows a stimulatory effect on TLR-2/4 expression via blocking the inhibitory cytokines and activating macrophages [29, 30]. Zymosan-like molecules or glucan compounds can induce release of IFN-γ in macrophages [23, 31]. Total crude extract of a strain of *Saccharomyces cerevisiae* composed of β-glucan, protein and lipid (similar to zymosan) could induce IL-10, IL-17 and IFN-γ at low dose [31]. Zymosan itself can induce CD3-activated thymocytes and splenocytes to produce IFN-γ [32].

In addition, activated macrophages have higher surface expression of TLR2 and 4 [29]. Activation of cell surface TLRs by ligand binding results in activation of both NF-κB-dependent and interferon regulating factor (IRF)-dependent signaling. Activation of these pathways leads to leukocyte recruitment and their immigration to sites of inflammation [33]. As a result, some anti-cancer therapies based on TLR-signaling are under development [8, 34].

Stimulation of TLRs-2, − 3 and − 4 results in the production of pro-inflammatory cytokines such as TNFα, IL-1, IL-6 and G-CSF and chemokines [35]. These inflammatory mediators play an important role in tumor rejection by activating T cells [34]. For example, TNF-α enhances leukocyte recruitment to inflammatory sites and promotes infiltration of cytotoxic T cells into the tumor vicinity [8] and TLR2-mediated production of IL-12 and TNF-α by macrophages results in NK cell activation and induction of the adaptive immune response [36, 37]. OM-174 a TLR-2/4 agonist together with BCG demonstrated anti-cancer activity through induction of TNF-α [9], whilst krestin, a selective TLR-2 agonist, inhibits breast cancer in mice [10]. In our study, zymosan treatment significantly increased TLR-2 and TNF-α gene expression in macrophages and enhanced serum TNF-α levels. This was in direct contrast to the suppression of these genes by the presence of melanoma alone.

Beside the generally accepted apoptosis-inducing and anti-tumor activity of TNF-α, it can also promote metastasis of tumor cells by inducing the chemokine receptors on the tumor cells particularly in late stage cancers [37]. However, recent studies have demonstrated the efficacy of TNF-α against melanoma [7, 38, 39]. TNF-α induces extravasation of erythrocytes and lymphocytes ending to hemorrhagic necrosis [37]. Moreover, TNFα targets tumor-associated vasculature by elimination of vascular lining and induction of hyper-permeability within tumor environment. These effects then provide better permeability of cytostatic drugs into the tumor milieu [37]. Recently, 5-aza an apoptosis-inducing agent with promising anti-melanoma effects was shown to act through the induction of TNF-α in melanoma tumor cells leading to caspase-dependent apoptosis [38]. Intra-tumoral administration of L19-TNF-α, a combination anti-cancer therapy, showed significant anti-melanoma activity in a phase II clinical trial in patients with advanced stage metastatic melanoma [39]. The clinical effect was associated with TNF-α-mediated extravasation of leukocytes, necrosis, hemorrhage and infiltration of cytotoxic CD8+ T cells into the tumor site [39]. Also, intra-tumoral administration of adenoviruses encoding IL-2 and TNF-α into B16 melanoma tumors showed anti-tumor activity in comparison to T-cell or control viral transfer alone [7].

Glucans and zymosan could exhibit a stimulatory and mitogenic effect on cytotoxic T cells such as NK cells, CD8+ T and macrophages [36, 40]. Zymosan at the doses between 1 and 100 μg/mL is mitogenic for rat thymocytes [41]. In a recent study zymosan stimulated the adaptive immune system by increasing peritoneal lymphocyte numbers as well as causing the proliferation of peripheral blood T- and B-cells [42]. Our results showing a mitogenic/proliferative activity of zymosan on murine splenocytes ex-vivo are in agreement with these findings. We observed a bell-shaped concentration-response curve on lymphocyte proliferation and viability. The reduction on proliferation and viability at the highest concentrations might reflect zymosan toxicity for lymphocytes at the highest concentrations studied.

Glucans also stimulate the production of interferons that activate NK cells which can directly kill tumor cells [36]. In addition, particulate β-glucans, like those in insoluble glucan part of zymosan, can prime cytotoxic T-lymphocytes and stimulate Th1 responses [43]. Zymosan particles were phagocytized by macrophages and changed their morphology to an activated form which produce more cytokines and promote T-cell activation and migration [36, 44]. Zymosan may also activate neutrophils through the CR3 receptor and induce superoxide production. Pre-treatment of neutrophils with TNF-α stimulates phagocytosis activity and superoxide production in response to activation by zymosan [36].

## Conclusion

The success of a cancer immunotherapy is largely dependent upon the patient’s immune system, tumor progression stage and the method, route and administration site. We evaluated TLR2, TLR4 and TNF-α mRNA expression in peritoneal macrophages and serum TNF-α protein levels at a single time-point after tumor induction and after therapeutic intervention with zymosan. Further studies on the dose- and time-course of zymosan anti-tumor effects and on its mechanism of action are required. Since intact zymosan is insoluble, it is important to either increase zymosan solubility or use more active components of zymosan that may require lower therapeutic doses to enhance the effective biological activity of zymosan. These limitations notwithstanding, our results indicate that zymosan effectively attenuates melanoma presence when dosed therapeutically in an animal model and may therefore be useful as an alternative or complementary therapy. This effect was associated with immune reprogramming but further studies are required to explore the exact effects of zymosan on immune cells activation.

## Abbreviations

CR3: Complement receptor 3
DMEM: Dulbecco’s Modified Eagle’s Medium
FBS: Fetal bovine serum
G-CSF: Granulocyte colony-stimulating factor
LPS: Lipopolysaccharide
MTT: 3-(4,5-dimethylthiazol-2-yl)-2,5-diphenyltetrazolium bromide
NF-κB: Nuclear Factor Kappa Beta
NK cells: Natural killer cells
PAMPs: Pathogen associated molecular patterns
RPMI: Roswell Park Memorial Institute
TLR: Toll-like receptor
TLR-2: Toll-like receptor 2
TNF-α: tumor necrosis factor α
